# The Effects of Photobiomodulation of 808 nm Diode Laser Therapy at Higher Fluence on the *in Vitro* Osteogenic Differentiation of Bone Marrow Stromal Cells

**DOI:** 10.3389/fphys.2018.00123

**Published:** 2018-02-23

**Authors:** Andrea Amaroli, Dimitrios Agas, Fulvio Laus, Vincenzo Cuteri, Reem Hanna, Maria Giovanna Sabbieti, Stefano Benedicenti

**Affiliations:** ^1^Department of Surgical and Diagnostic Sciences, Laser Therapy Center, University of Genoa, Genoa, Italy; ^2^School of Biosciences and Veterinary Medicine, University of Camerino, Macerata, Italy

**Keywords:** laser therapy, low-level-laser therapy, phototherapy, stem cells, bone, anti-inflammatory effect, differentiation, cytokines

## Abstract

The literature has supported the concept of mesenchymal stromal cells (MSCs) in bone regeneration as one of the most important applications in oro-maxillofacial reconstructions. However, the fate of the transplanted cells and their effects on the clinical outcome is still uncertain. Photobiomodulation (PBM) plays an important role in the acceleration of tissue regeneration and potential repair. The aim of this *in vitro* study is to evaluate the effectiveness of PBM with 808 nm diode laser therapy, using a flat-top hand-piece delivery system at a higher-fluence (64 J/cm^2^) irradiation (1 W, continuous-wave) on bone marrow stromal cells (BMSCs). The BMSCs of 3 old female Balb-c mice were analyzed. The cells were divided into two groups: irradiated group and control group. In the former the cells were irradiated every 24 h during 0 day (T0), 5 (T1), 10 (T2), and 15 (T3) days, whereas the control group was non-irradiated. The results have shown that the 64 J/cm^2^ laser irradiation has increased the Runt-related transcription factor 2 (Runx2). Runx2 is the most important early marker of osteoblast differentiation. The higher-fluence suppressed the synthesis of adipogenic transcription factor (PPARγ), the pivotal transcription factor in adipogenic differentiation. Also, the osteogenic markers such as Osterix (Osx) and alkaline phosphatase (ALP) were upregulated with an increase in the matrix mineralization. Furthermore, western blotting data demonstrated that the laser therapy has induced a statistically valid increase in the synthesis of transforming growth factor β1 (TGF-β1) but had no effects on the tumor necrosis factor α (TNFα) production. The data has statistically validated the down-regulation of the important pro-inflammatory cytokines such as interleukin IL-6, and IL-17 after 808 nm PBM exposition. An increase in anti-inflammatory cytokines such as IL-1rα and IL-10 was observed. These *in vitro* studies provide for first time the initial proof that the PBM of the 808 nm diode laser therapy with flat-top hand-piece delivery system at a higher-fluence irradiation of 64 J/cm^2^ (1 W/cm^2^) can modulate BMSCs differentiation in enhancing osteogenesis.

## Introduction

Within dentistry, tissue regenerative concepts have been developed to be applied in the clinical areas of periodontology and implantology (Izumi et al., 2003; Egusa et al., 2012). Oro-maxillofacial reconstruction procedures can assist in facial tissue repair and regeneration, such as may be the result of trauma or neoplasm. In Europe, about 1.5 million patients undergo craniofacial reconstruction procedures annually. However, ~20% of them continue to experience functional deficiencies despite the surgical interventions. Furthermore, about 30,000 patients per year would develop donor-site morbidity following oral and maxillofacial reconstruction (Czerwinski et al., 2010; Rodriguez y Baena et al., 2016).

The research area of utilizing stem cells toward restoring the structure and function of damaged tissues or organs in medicine and dentistry (Huang et al., 2009; Meirelles Lda and Nardi, 2009) is moving fast and attracting the interest of researchers. Many studies have shown that the use of mesenchymal stromal cells (MSCs) for bone regeneration is one of the most important applications in oro-maxillofacial reconstruction (Egusa et al., 2012; Zigdon-Giladi et al., 2014), periodontology and implantology (Izumi et al., 2003; Egusa et al., 2012). Despite the positive results that were achieved in bone regeneration through utilizing the MSCs, the destiny of the transplanted cells and their effect on the clinical outcome remains uncertain. This could be related to the fact that the transplanted cells die very quickly or migrate out of the transplantation site according to several animal studies (Meijer et al., 2008; Zimmermann et al., 2011; Egusa et al., 2012). Therefore, the challenge of stem cell research is related to the identification of supporting therapies, which can improve and enhance the effectiveness of various tissues repair and regeneration.

Utilization of a low-energy light intensity within the visible red and near infrared portion of the electromagnetic spectrum has been shown to stimulate irradiated cellular activity (Avci et al., 2013). This phenomenon has been referred to as photobiomodulation (PBM) (Avci et al., 2013; Amaroli et al., 2015a). Many cellular responses have been observed in *in vitro* models after irradiation with light sources of different wavelengths at specific energy density, which has induced PBM effects. These cellular responses resulted in: an increase of mitochondrial respiration and ATP production (Karu, 2010; Amaroli et al., 2015b,c, 2016c), the synthesis of proteins (Avci et al., 2013; Lipovsky et al., 2013) and in cells migration and proliferation (Avci et al., 2013; Amaroli et al., 2015a). Furthermore, reduction in the inflammation, acceleration of wound healing (Avci et al., 2013; Amaroli et al., 2018) and bone formation (Santinoni et al., 2017) were observed. Few studies have investigated the use of PBM on MSCs' stimulation (Lipovsky et al., 2013; Kushibiki et al., 2015). These studies have pointed out that the effectiveness of PBM on MSCs was related to enhanced angiogenic effects of adipose-derived stromal cells (Park et al., 2014) and to the modulation of gene expression, proliferation, intracellular cAMP levels, and osteogenic differentiation (Kushibiki et al., 2015). However, despite *in vivo* studies (animal models and randomized controlled clinical trials) with a positive PBM outcome, it remains a controversial subject as a consequence of conflicting effects produced by various operating parameters such as wavelength, fluence, laser power output, exposure time, number of applications (Jenkins and Carroll, 2011), and the beam profile (Amaroli et al., 2016b).

Recently, the AB2799 flat-top hand-piece has been developed and marketed. It can generate a more homogenous irradiation at ~1 cm^2^ (Spot size) from contact to 105 cm distance from the target tissue (Amaroli et al., 2016b; Selting, 2016). This probe allows the use of relatively higher power densities and fluences with lower risk of causing collateral thermal damage to adjacent tissues, in comparison to standard (Gaussian profile) hand-pieces (Amaroli et al., 2015a,b; Selting, 2016). Previous studies by Amaroli and co-workers have shown that, the use of the 808 nm diode laser with the flat-top hand-piece at a higher-fluence of 64 J/cm^2^ and power output of 1 Watt (W) in continuous-wave (CW) emission, resulted in increased mitochondrial activity (Amaroli et al., 2016b), in stimulation of oxygen consumption (Amaroli et al., 2015b) and in ATP production (Amaroli et al., 2015c, 2016c) in the unicellular organism *Paramecium primaurelia*. In the same unicellular model, modulation of calcium fluxes and intracellular calcium concentration (Amaroli et al., 2015c, 2016a) was also observed, as well as an increment of fission rate rhythm (Amaroli et al., 2015a). In addition, it was evidenced that this higher-fluence did not generate genotoxic damage or adverse effects (Amaroli et al., 2017) while it was able to induce anti-inflammatory effects that promoted the wound healing in an animal model (Cuteri et al., 2016; Amaroli et al., 2018).

The purpose of this study was to assess the *in vitro* ability of 808 nm diode laser therapy with flat-top hand-piece delivery system at a higher-fluence and -power (64 J/cm^2^; 1 W, 1 W/cm^2^) on inducing osteoblast maturation and on modulating the bone marrow stromal cells (BMSCs) secretion of important inflammatory mediators.

Latterly, increasing findings point to the properties of BMSCs; a cell population isolated from the bone marrow stroma and constituted by multiple cell types including multipotent progenitors for skeletal lineages (Bianco and Robey, 2015). In addition to their ability to differentiate into skeletal tissue cells, the BMSC possess immunomodulatory characteristics (Kuroda et al., 2017). The current research is increasingly demonstrating the importance of the interaction of the bone marrow stem/progenitor and the mature cell in their role in bone metabolism, turnover, and regeneration (Agas et al., 2015; Kassem and Bianco, 2015). Within this investigation, the BMSCs of 3 month old female Balb-c mice were analyzed through histochemical staining (alkaline phosphatase and alizarin red S assays), immunoassay (cytokines and chemokines assays) and western blotting (expression of runt-related transcription factor 2, osterix, peroxisome proliferator-activated receptor gamma, transforming growth factor β1, tumor necrosis factor α).

## Materials and methods

### Laser unit

In this study, a diode laser (λ 808 nm) (Doctor Smile–LAMBDA Spa–Vicenza, Italy) with the AB2799 hand-piece was utilized to irradiate the BMSCs. According with the technical data (http://www.doctor-smile.com/assets/prodotti/accessori/pdf/ST_FLATTOP_EN.pdf), the AB2799 hand-piece with a flat-top profile delivers more homogenous irradiation over ~1 cm^2^ surface area and has the same irradiation spot area and energy from contact to 105 cm of distance from the target tissue in comparison to the standard hand-piece, which delivers Gaussian profile irradiation (Amaroli et al., 2016b; Selting, 2016).

### Bone marrow stromal cells (BMSCs) derivation

All the described animal-related procedures were conducted according to Directive 2010/63/EU of the European Parliament and of the Council of 22nd September 2010 on the protection of animals used for scientific purposes (Article 3, Paragraph 1), the Italian Legislation (D. Lgs. n. 26/2014, Article 3, Paragraph 1, Letter a), which does not require any approval by the competent authorities. Three month old female Balb-c mice (Harlan Italy SrL, Correzzana, Milano, Italy) were used. The mice were kept in a laminar-flow cage in a standardized environmental condition. Food (Harlan, Italy) and water were supplied *ad libitum*. The mice were sacrificed by cervical dislocation.

Long bones (femurs, tibiae, and humeri) were dissected to be free from any adhering tissues. Bone ends were removed and the marrow cavity flushed out. Cells were pooled and plated on 100 mm culture dishes. Cells were grown in Roswel Park Memorial Institute (RPMI) culture medium added with 10% heat-inactivated-fetal calf serum (HIFCS), penicillin (100 U/ml), and streptomycin (50 μg/ml) (all from Invitrogen Life Technologies, Milan, Italy) for 10 days at 37°C in a humidified atmosphere of 5% CO_2_ in order to generate monolayers of non-hematopoietic adherent cells (Bianco et al., 2013) (referred as “BMSCs”). The culture medium was replaced every 3 days.

### BMSCs cultures for the experimental studies

Cells were detached using 0.25% trypsin for 2 min at room temperature and plated as follow: for the western blotting and the cytokines and chemokines assays (sections Western Blotting and Cytokines and Chemokines Assay, respectively), the BMSCs were plated on a 24-well culture plate at the density of 10 × 10^5^ cells/well; for Alkaline Phosphatase assay and Alizarin Red S histochemical staining (section Alkaline Phosphatase Assay and Alizarin Red S Histochemical Staining) the BMSCs were plated on six well culture plates at the density of 10 × 10^5^ cells/well; for the immunolabeling of Run-related transcription factor 2 (Runx2) or osterix (Osx)(section Single Immunolabeling) the BMSCs were plated at the density of 10 × 10^4^ cells/well on round coverslips, which previously sterilized and inserted in the six well culture plates.

Cells were plated in RPMI culture medium with added HIFCS, except for the cultures used for Alkaline Phosphatase (ALP) assay and Alizarin Red S staining (section Alkaline Phosphatase Assay and Alizarin Red S Histochemical Staining) that were plated in osteogenic medium (RPMI, 10% HIFCS, 8 mM β-glycerophosphate, and 50 μg/ml ascorbic acid). The same culture conditions were provided during the conduction of the experiments.

One day after cell seeding (passage 1), all the cultures were irradiated with 808 nm wavelength laser or untreated as below described. Culture medium was changed every 3 days.

### Irradiation parameters

The culture dishes (Table 1A) were uncovered and then wrapped up in the aluminum foils (Table 1B) with a hole (Tables 1C,D) of a diameter corresponding to the diameter of the laser spot area of the hand-piece to avoid energy dispersion (Johnstone et al., 2014). The cultures were then irradiated using the 808 nm diode laser with the AB2799 flat-top hand-piece delivery system (Table 1E). The laser irradiation was performed with the hand-piece in contact with the aluminum foil hole at 1 cm distance from the cells.

**Table 1:** Description of the irradiation parameters and the experiments' design.

**IRRADIATION PARAMETERS**				
**Laser**	**Wavelength**	**Hand-piece**	**Electromagnetic wave**	**Power**
Diode	808 nm	Flat-top	Continuous wave	1 W
**Irradiation time**	**Fluence**	**Irradiance**	**Distance**	**Treatments**
60 s	64 J/cm^2^	1 W/cm^2^	1 cm	Every 24 h for 0 days, 5 days, 10 days and 15 days
**EXPERIMENTAL DESIGN**				
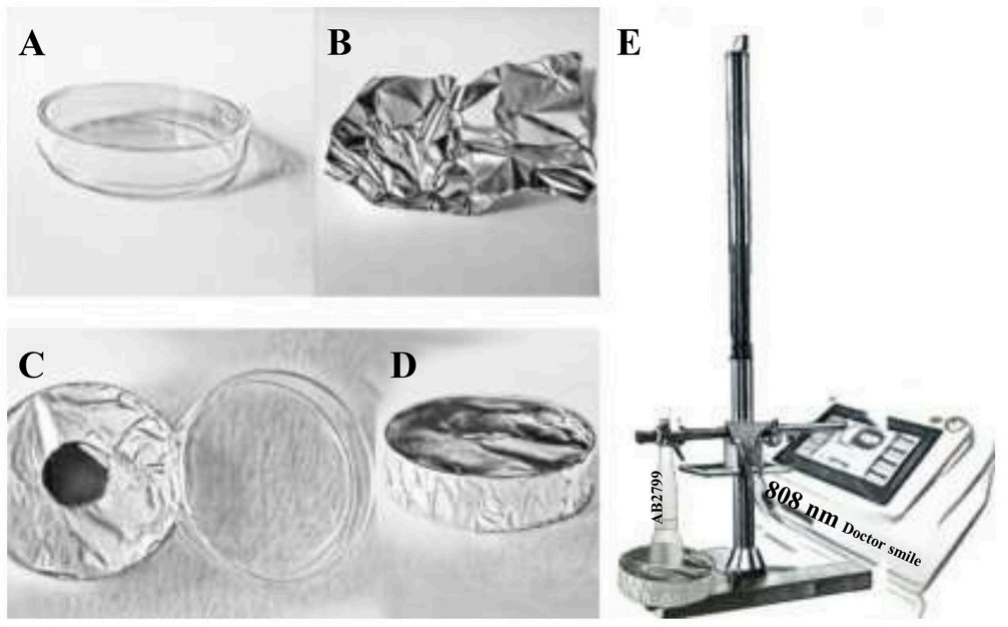				

The cells were irradiated with 1 W in CW (power density 1 W/cm^2^) for 60 s of irradiation exposure time to generate a final fluence of 64 J/cm^2^. This application of laser irradiation was repeated every 24 h over different periods of time (Day 0: T0; 5 days: T1; 10 days: T2; 15 days: T3). The related control cultures (Day 0: C0; 5 days: C1; 10 days: C2; 15 days: C3) were maintained in identical conditions except that the laser device was switched off.

### Western blotting

The proteins from irradiated BMSCs or untreated (control) cells were extracted in cell lysis buffer (Cell Signaling, EuroClone) at the end of each set of irradiation (Day 0, 5 days, 10 days, 15 days) and the concentration was determined by the bicinchoninic acid protein assay reagent (Pierce, EuroClone).

The Western blotting was performed as previously described (Agas et al., 2016). Membranes were immunoblotted in blocking buffer with specific antibodies: rabbit anti-runt-related transcription factor 2 (Runx-2) antibody (1:800 dilution, Cell Signaling, Euroclone, Milano, Italy); rabbit anti-Osterix (Osx), rabbit anti-peroxisome proliferator-activated receptor gamma (PPARγ) antibody (1:600 dilution, Santa Cruz Biotechnology—DBA, Milano, Italy); rabbit anti- transforming growth factor β 1 (TGF-β1) antibody (1:600 dilution, Abcam, Prodotti Gianni, Milano, Italy); rabbit anti-tumor necrosis factor α (TNFα) (1:500 dilution, BioLegend, Microtech SrL, Napoli, Italy). After washing blots, they were incubated with horseradish peroxidase (HRP)-conjugated donkey anti-rabbit immunoglobulin G (IgG) or with HRP-conjugated rabbit anti-mouse IgG (Cell Signaling, Euroclone Milano, Italy). The immunoreactive bands were visualized using luminol reagents/ECL film according to the manufacturer's instructions. In order to normalize the bands, filters were stripped and re-probed with a monoclonal anti-α-tubulin (Sigma-Aldrich, Milano, Italy). The bands' density was densitometrically quantified by NIH Image.

### Single immunolabeling

At the end of each set of irradiations, the untreated and irradiated BMSCs were fixed in 4% PFA and permeabilized with 0.3% Triton X-100 as previously described (Sabbieti et al., 2010). The cultures were then incubated for 2 h at room temperature with the following primary antibodies: rabbit anti-runt-related transcription factor 2 (Runx-2) antibody (1:100 dilution, Cell Signaling, Euroclone, Milano, Italy) or rabbit anti-Osterix (Osx) antibody (1:50 dilution, Santa Cruz Biotechnology—DBA, Milano, Italy). After washing the cells with PBS, they were incubated with Alexa Fluor-488 chicken anti-rabbit IgG or with Alexa Fluor 594 goat anti-rabbit IgG (both 1:100 dilution, Life Technologies, Monza, Italy) for 1 h at room temperature. The reaction controls were performed by complexing the primary antibody with a relative blocking peptide or by omitting the primary antibody. Coverslips were mounted on slides with PBS/glycerol (1:1). The slides were imaged using fluorescent microscopy on Zeiss Axioplan microscopy. Fluorescence analysis was performed by a fluorimeter Tecan Infinite with excitor filter 590 nm and emission 635 nm for Alexa Fluor 594, or 485, and of 535 nm for Alexa Fluor 488. A Tecan Infinite fluorescence reader quantified the amount of Alexa Fluor 594-labeled anti-Osx and Alexa Fluor 488-labeled anti-Runx2.

### Alkaline phosphatase assay and alizarin red S histochemical staining

The cells maintained in osteogenic medium (Naganawa et al., 2006, 2008) were irradiated or untreated as described above in section BMSCs Cultures for the Experimental Studies. At the end of each set of irradiations (Day 0, 5 days, 10 days, 15 days), the cells were fixed in 4% paraformaldehyde (PFA) for 20 min at room temperature. The alkaline phosphatase (ALP) staining was performed with a commercial kit (Sigma-Aldrich) according to the manufacturer's instructions. NIH Image has measured the ALP colonies. For the Alizarin Red S staining, the untreated and irradiated cells were stained to assess the mineralized matrix as previously described (Sabbieti et al., 2013). The cells' layers were briefly rinsed with PBS and fixed in 4% PFA. Then, the cultures were stained with 2% Alizarin Red S (pH 7.2, Sigma-Aldrich) for 20 min at 37°C. The cultures were examined under light microscopy (Zeiss Axioplan; Zeiss S.p.A., Milano, Italy). The stain was desorbed and the collected solutions were distributed as 100 μL/well on 96-well plates for absorbance reading at 590 nm by spectrophotometry (Tecan Infinite reader; Tecan, Milano, Italy).

### Cytokines and chemokines assay

The cytokine/chemokine profiles in supernatants of the cultured BMSCs population in the laser irradiated group or untreated group (control) were assessed at the end of each set of irradiation (Day 0, 5 days, 10 days, 15 days) by using Mouse Cytokine Array Panel A kit (R&D Systems, Milano, Italy) according to the manufacturer's instructions as previously described (Sabbieti et al., 2015).

### Statistical analysis

Data were analyzed by using one-way ANOVA followed by Tukey's pairwise comparisons. The letters a, b, c, or d above a particular column indicate statistically significant difference for that group in comparison to another group within a particular graph. Model *p*-value and sample number labeled, with standard error depicted for each treatment site. Values of ^*^p < 0.05 were considered significant.

The significance of difference between two groups was evaluated with an unpaired two-tailed Student's *t-*test and differences were considered significant at ^*^p < 0.05.

The results are a representative of those obtained by independent experiments repeated at least three times (samples per group *n* = 3).

## Results

### Effects of photobiomodulation on osteoblast differentiation

**Figure 1:**
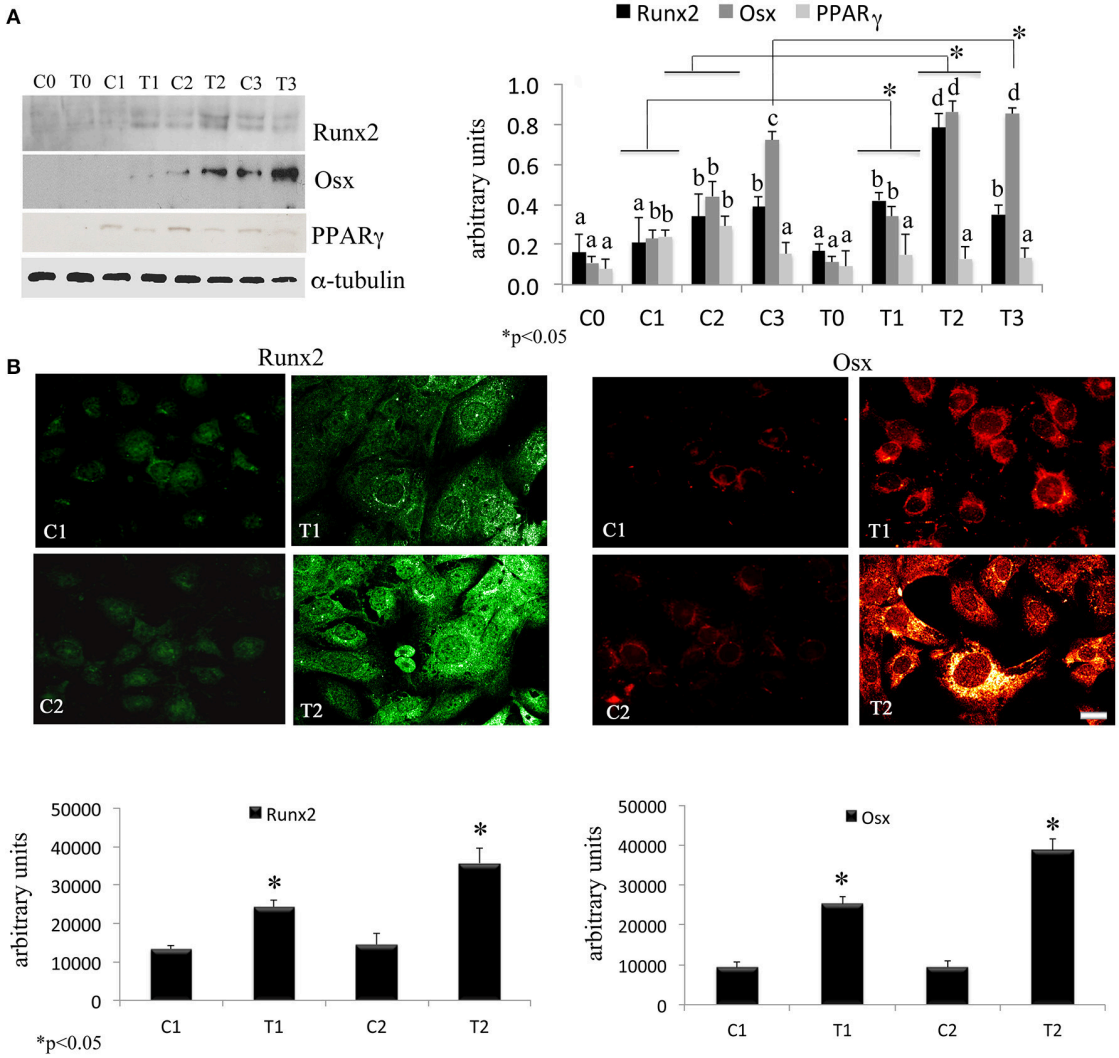
**(A)** Time-course effects of laser irradiation on Runx2, osterix, and PPARγ synthesis in BMSCs by western blotting analysis. Cells were laser irradiated over different period of time (Day 0: T0; 5 days: T1; 10 days: T2; 15 days: T3). Control cultures (Day 0: C0; 5 days: C1; 10 days: C2; 15 days: C3) were maintained in identical conditions except that the laser device was switched off. At the end of each treatment, proteins from BMSCs irradiated and untreated were extracted, subjected to SDS-PAGE, transferred to a PVDF membrane, and probed with rabbit anti-Runx2, rabbit anti-osterix, or rabbit anti-PPARγ antibodies; filters were stripped and re-probed mouse anti-α-tubulin antibody to show equal amount of loading. Graphic represents results of three independent experiments. Data were analyzed by using one-way ANOVA. Lowercase letters denote homogeneous subsets. Error bars represent ± SE (^*^p < 0.05) **(A)**. The labeling pattern of Runx2 or osterix in BMSCs laser irradiated and untreated. Representative images of Runx2 or osterix localization using a rabbit anti-Runx2 antibody (green: Alexa Fluor 488 staining) and the rabbit anti-osterix antibody (red: Alexa Fluor 594 staining). Bar, 50 μm. Fluorescence analysis from a pool of three different experiments was quantified by a Tecan Infinite fluorescence reader. The values that analyzed by Magellan v4.0 software are reported as means ± SD and statistically analyzed; ^*^p < 0.05 **(B)**.

**Figure 2:**
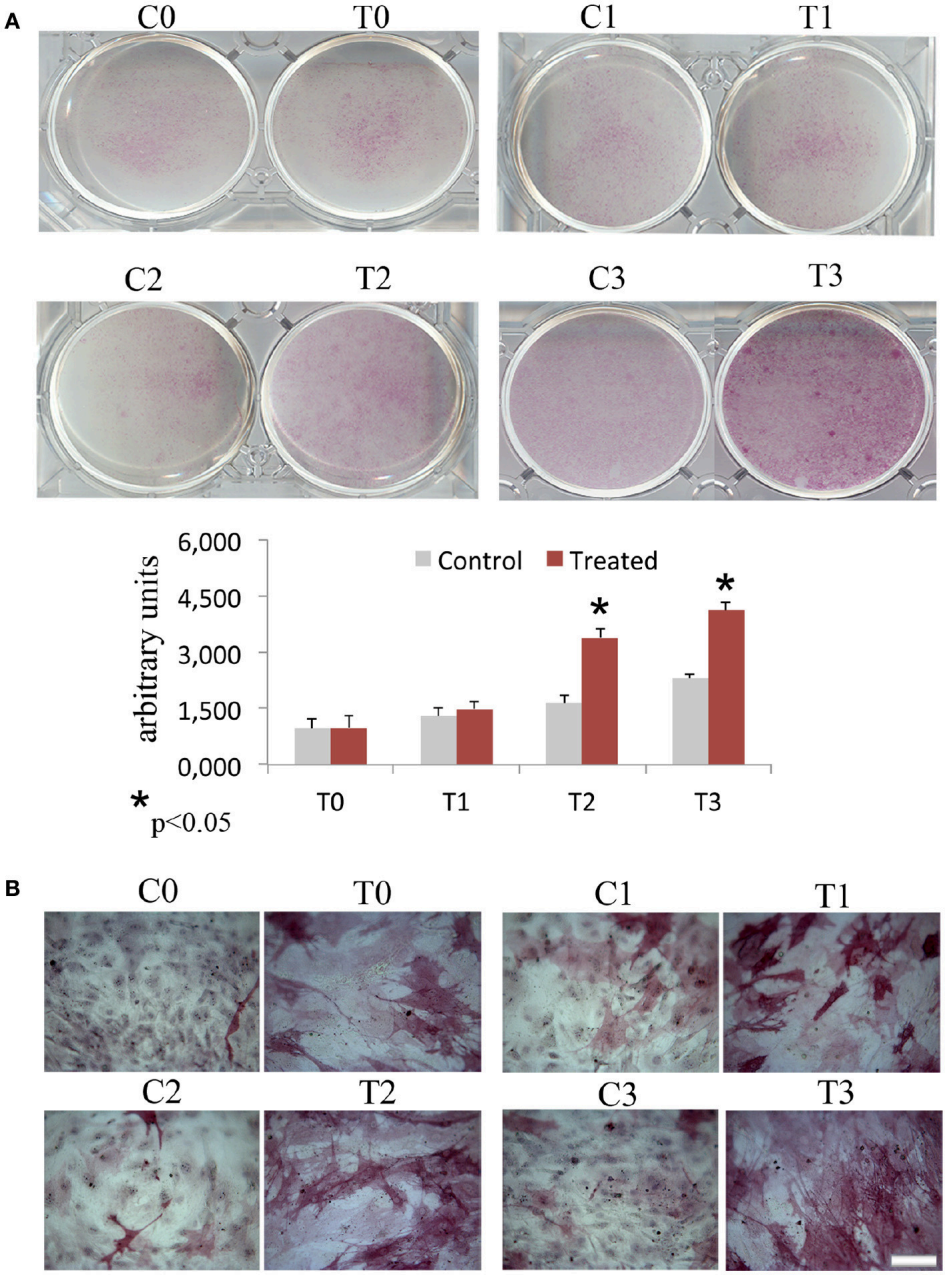
Time-course effects of laser irradiation on ALP activity. BMSCs grown in osteogenic medium, as detailed in Materials and Methods section, was laser irradiated over different periods of time (Day 0: T0; 5 days: T1; 10 days: T2; 15 days: T3). Control cultures (Day 0: C0; 5 days: C1; 10 days: C2; 15 days: C3) were maintained in identical conditions except that the laser device was switched off. At the end of each treatment, colony area was measured by NIH Image both in cultures laser irradiated and in the untreated ones. The values are reported as means ± SD and statistically analyzed; ^*^p < 0.05. vs. the corresponding untreated BMSCs **(A)** were in accordance with ALP staining observed with a light microscope. Bar, 100 μm **(B)**.

**Figure 3:**
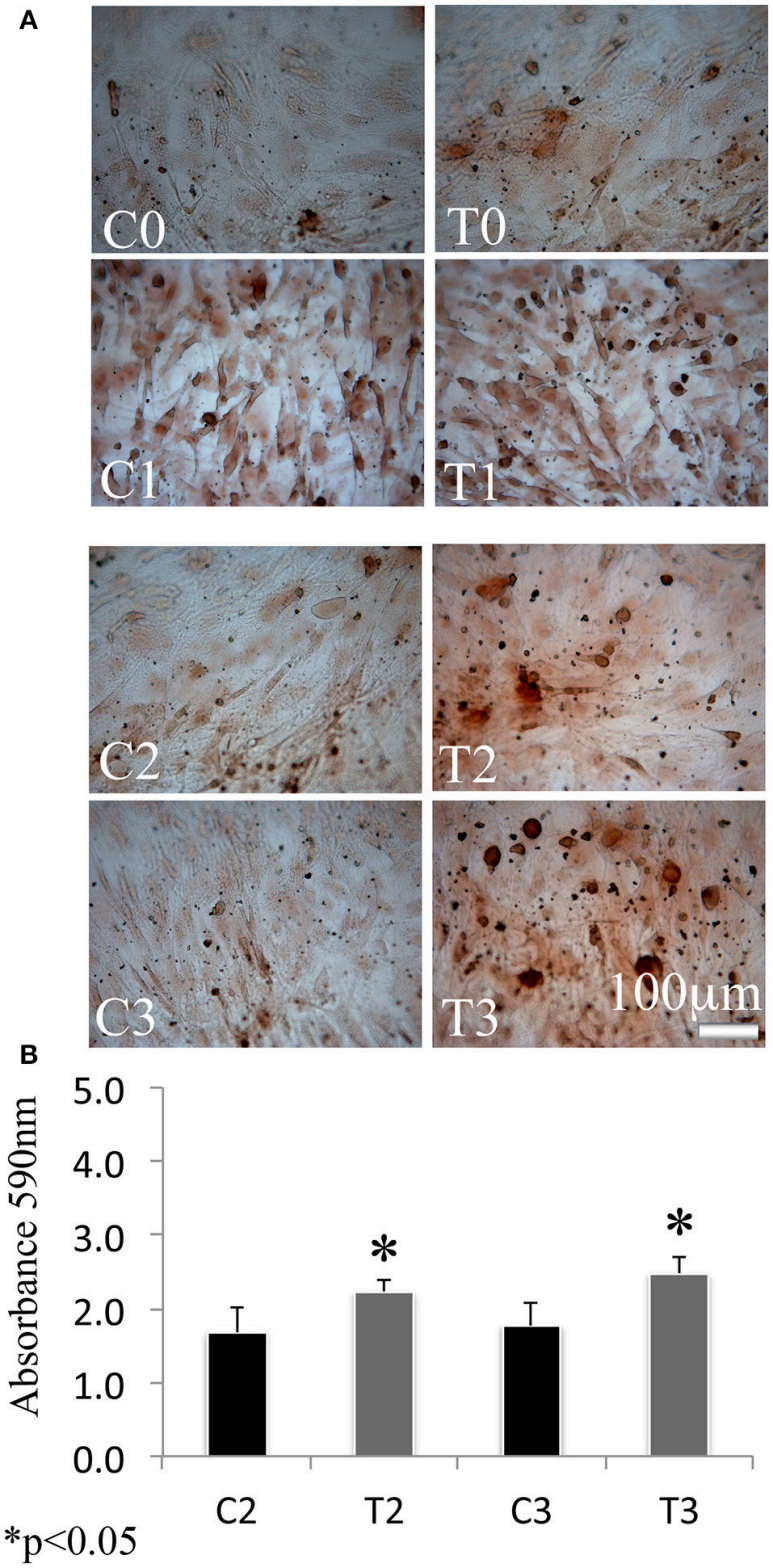
Time-course effects of laser irradiation on calcium deposition. BMSCs grown in osteogenic medium, as detailed in the Materials and Methods section, were laser irradiated over different period of time (Day 0: T0; 5 days: T1; 10 days: T2; 15 days: T3). Control cultures (Day 0: C0; 5 days: C1; 10 days: C2; 15 days: C3) were maintained in identical conditions except that the laser device was kept off. The results show the Alizarin red S staining observed with a light microscope (Bar, 100 μm) **(A)** and are in accordance with quantitative analysis of Alizarin red staining in which the values are reported as means ± SD and statistically analyzed; ^*^p < 0.05. vs. the corresponding untreated BMSCs **(B)**.

In order to assess the effects of laser therapy on osteoblast maturation, BMSCs were treated with PBM therapy at different time points, which corresponded at 0 (T0), 5 (T1), 10 (T2), and 15 (T3) days. The Western blotting analyses of laser treated cells indicated a significant increase of both Runx2 and Osx transcription factors that started at the time point T1 and reached maximal level at the time point T2 (Figure 1A). At the time point T3 the increase of Osx was maintained, while Runx2 synthesis was decreased. Notably, a decrease of the adipogenic transcription factor PPARγ was evident (Figure 1A). In addition, the findings from *in situ* investigations to examine the subcellular distribution of Runx2 and Osx showed an increase of the Runx2 and the Osx labeling at cytoplasm and perinucleare level in irradiated BMSCs at time point T1. Interestingly, the fluorescence analysis at the time point T2 revealed a strong accumulation of the mean fluorescence intensity for Runx2 (247.17% vs. untreated control) and osterix (380.43% vs. untreated control) particularly at perinuclear and nuclear level (Figure 1B). This evidence indicates the correct trafficking of Runx2 and Osx to the nucleus. This process is required for their function as regulators of bone formation gene expression. No differences were found at T0 time point between laser-treated and untreated cells (data not shown). Since the alkaline phosphatase (ALP) is an important marker of osteogenic differentiation, we examined the effects of laser on ALP positive colonies formation. At 10 days of the treatment, an increase of ALP positive colonies was observed and its maximum number was reached after 15 days (Figures 2A,B). Additionally, the Alizarin Red S staining was used to measure the calcium deposition that is considered as a late differentiated marker; a notable increase of calcium deposition was found after 15 days of laser treatment (Figures 3A,B).

### Effects of low-level-laser therapy on the cytokines synthesis and release by BMSCs

Predominantly, stromal stem/progenitor and mature cells release the signaling molecules which are released within bone marrow reservoir. They exert pivotal roles in regulating bone remodeling, in particular the transforming growth factor β1 (TGF-β1), which is involved in bone formation.

It is worth noting that TNFα is involved in inflammatory bone erosion.

**Figure 4:**
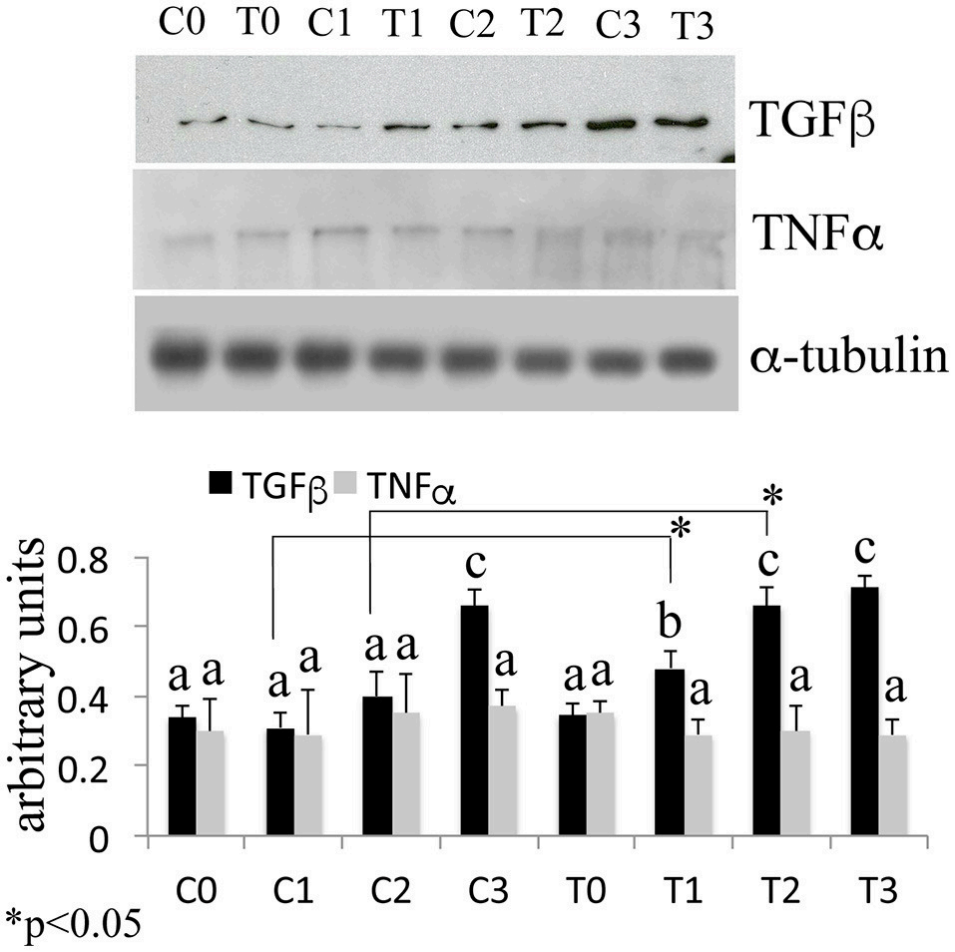
Time-course effects of laser irradiation on TGF-β1 and TNFα synthesis in BMSCs by western blotting analysis. Cells were laser irradiated over different periods of time (Day 0: T0; 5 days: T1; 10 days: T2; 15 days: T3). Control cultures (Day 0: C0; 5 days: C1; 10 days: C2; 15 days: C3) were maintained in identical conditions except that the laser device was switched off. At the end of each treatment, proteins from BMSCs irradiated and untreated were extracted, subjected to SDS-PAGE, transferred to a PVDF membrane, and probed with rabbit anti-TGF-β1 rabbit anti-TNFα; then, filters were stripped and re-probed mouse anti-α-tubulin antibody to show equal amount of loading. Graphic represents results of three independent experiments. Data were analyzed by using one-way ANOVA. Lowercase letters denote homogeneous subsets. Error bars represent ± SE (^*^p < 0.05).

**Figure 5:**
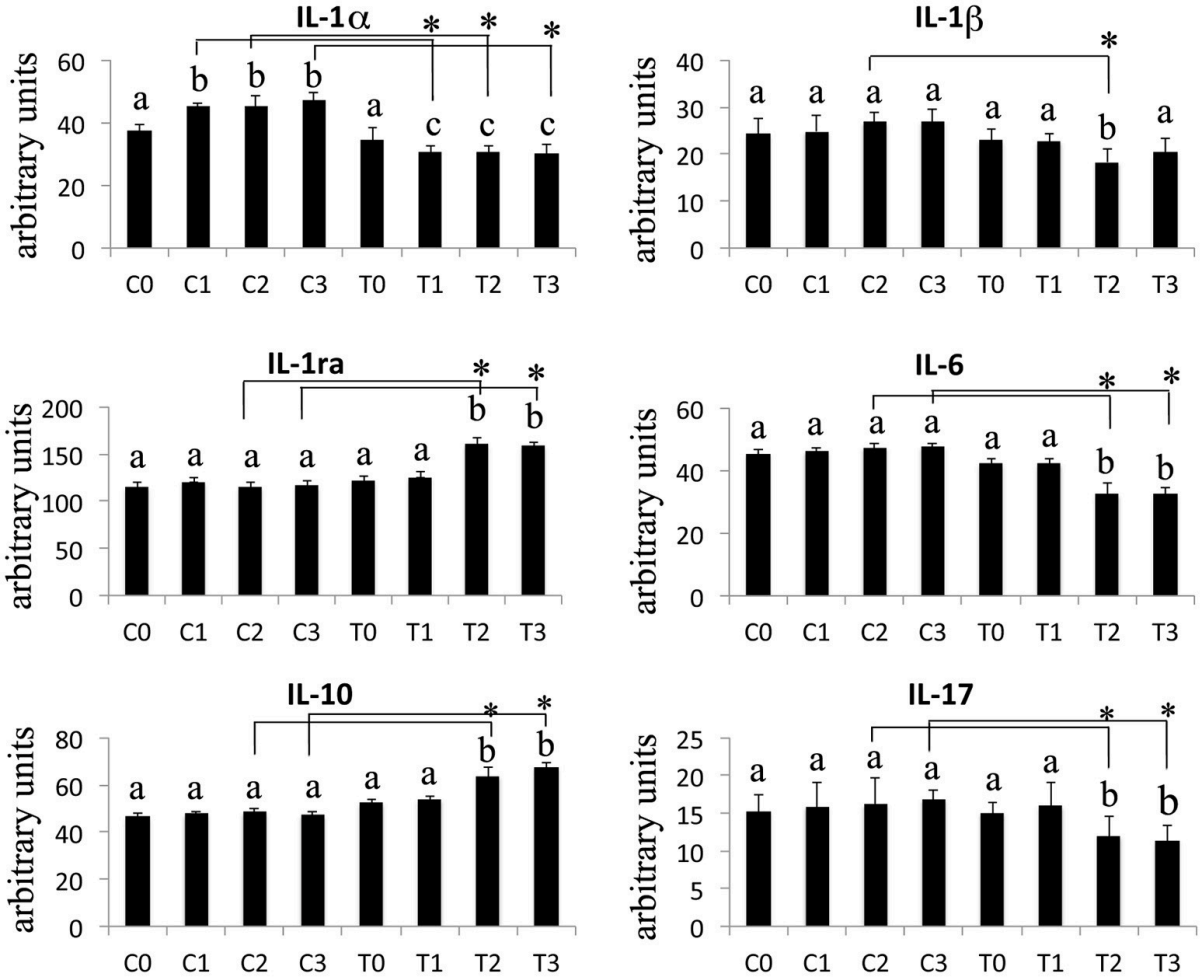
Effects of laser irradiation on cytokines and chemokines release. Cells were laser irradiated over different period of time (Day 0: T0; 5 days: T1; 10 days: T2; 15 days: T3). Control cultures (Day 0: C0; 5 days: C1; 10 days: C2; 15 days: C3) were maintained in identical conditions except that the laser device was switched off. At the end of each treatment cytokines and chemokines were analyzed in medium from BMSCs laser irradiated or untreated. Graphic represents results of three independent experiments. Data were analyzed by using one-way ANOVA. Lowercase letters denote homogeneous subsets. Error bars represent ± SE (^*^p < 0.05).

The Western Blotting data have demonstrated that the laser therapy had induced a statistically valid increase in the synthesis of TGF-β1 but had no effects on the TNFα production (Figure 4). However, the analysis of the BMSCs supernatants provided slight evidence but statistically validated the downregulation of the important pro-inflammatory cytokines such as interleukin (IL)-6, and IL-17 after laser irradiation. In addition, an increase in anti-inflammatory cytokines such as IL-1ra and IL-10 was observed (Figure 5).

## Discussion

Stem cell research is related to identifying optimal therapies that can be utilized in promoting tissue healing. Photobiomodulation could be a useful adjunctive, as it plays a role in acceleration of tissue regeneration and repair (Avci et al., 2013). Such application on stem cells may be therefore beneficial in many areas of biomedical application and ultimately, this would augment the success of regenerative medicine (Abrahamse, 2012). Our data appears to support this line of thought. In fact, it was found that the 808 nm laser irradiation at a fluence of 64 J/cm^2^ increased the BMSCs' Runx2, which is one of the early cell markers that promotes MSCs differentiation into immature osteoblasts and inhibits lineage development into adipocytes (Komori, 2010). Wu et al. (2012) study has shown that irradiation with gallium-aluminum-arsenide (GaAlAs) red laser (wavelength 660 nm) at different fluences of 1 J/cm^2^, 2 J/cm^2^, or 4 J/cm^2^ with power density 10 mW/cm^2^ can induce the generation of insulin-like growth factors 1 (IGF-1), to promote both the proliferation and the osteogenic differentiation of mouse bone marrow mesenchymal stem cells (D1 cells), whereas it may induce bone morphogenetic protein 2 (BMP2) expression primarily to enhance osteogenic differentiation. It is known that IGF-1 can regulate Runx2 DNA binding (Qiao et al., 2004; Guntur and Rosen, 2013) and BMP2 is able to induce osteoblast differentiation through Runx2-dependent activating transcription factor 6 (ATF6) expression (Jang et al., 2012). Therefore, our protocol of utilizing the 808 nm laser therapy at a higher-fluence can photobiomodulate the BMSCs in a way which is similar to what has been observed in Wu et al. (2013) results, when 660 nm was utilized to irradiate the cells at low-fluence. However, previous results by Bouvet-Gerbettaz et al. (2009) have failed to induce osteoblast differentiation with 808 nm wavelength at low-fluence (4 J/cm^2^). In contrast, Soleimani et al. (2012) has observed that 810 nm GaAlAs with range of fluences of 3–6 J/cm^2^ had a positive effect on the BMSCs differentiation. However, the author did not provide an explanation of the mechanism of action of the laser therapy.

In our study an induction of MSC to osteoblast precursors and an inhibition of their commitment to adipocytes lineage was demonstrated by the increase of Runx2 and the decrease of PPARγ (Komori, 2010) in culture exposed to irradiation for 5 (T1) and 10 days (T2). In addition, the differentiation of osteoblasts precursors into mature osteoblasts was demonstrated in laser-irradiated cells by the increase of Osx (Zou et al., 2006) overall, evident after 15 days (T3) of irradiation. At the same time point (T3) an enhanced ALP positivity and matrix mineralization (revealed by Alizarin red staining analysis) was found in irradiated cultures, compared to the untreated control cells. As anticipated, for the evaluation of matrix deposition both untreated (control) and treated cultures were grown on osteogenic medium to accelerate and increase the mineralization process, since cells growth in RPMI culture medium showed slow matrix deposition after laser treatment. In addition, the untreated cultures did not show any mineralization, in line with the fact that our experiments were conducted on a cell population involving overall undifferentiated cells.

Taken together, these data point to the fact that laser irradiation not only induced the MSCs development toward immature osteoblasts but also promoted osteoblast maturation.

It has been demonstrated that within the bone marrow, the process of MSCs maturation along osteoblastic lineage is finely regulated by signals, which are released in the marrow microenvironment. For instance, TGF-β1, besides its role in promoting osteoblastic precursors or matrix-producing osteoblasts through chemotactic attraction (Park et al., 2014), also blocks the apoptosis of osteoblasts (Huang et al., 2009) and enhances the osteoblast proliferation (Horwitz et al., 2002). If it is considered that our laser irradiation of BMSCs has no effect on the anti-proliferative and pro-apoptotic TNF-α protein, we would speculate that our therapy might maintain the cell proliferation process. This assumption has been supported by our previous results which have shown that laser therapy has positive effects on the protozoa and mammalian mitochondria activities (Amaroli et al., 2015b,c, 2016b) and stimulates the cell proliferation (Amaroli et al., 2015a) without inducing cellular damage (Amaroli et al., 2017). Furthermore, the literature has supported the fact that MSCs proliferation can be induced after laser irradiation (Abramovitch et al., 2005; Hou et al., 2008; Soleimani et al., 2012; Wu et al., 2012; Giannelli et al., 2013). In addition, it has been demonstrated that elevated levels of TGF-β1 play an important role in downregulating the release of the proinflammatory cytokine such as IL-6 and IL-17, thus promoting favorable conditions for bone regeneration (Zhou et al., 2008).

In laser treated BMSCs, we have consistently observed an increase in the synthesis of TGF-β1 associated with a decrease in the secretion of the pro-inflammatory molecules Il-1β, IL-6, and IL-17. Furthermore, the production of the anti-inflammatory interleukins IL-1ra, IL-10 was increased.

Although the changes in the cytokine levels were slight, it should be considered that the BMSCs used in these experiments were derived from healthy mice with a bone marrow environment under steady-state condition. Therefore, such small fluctuations in cytokines release may be interpreted as specific responses to the treatment.

Moreover, our data has showen that utilization of a higher-fluence at a higher-power setting has confirmed the antiflogistic propriety which has coincided with the findings of the wound healing model study in muscle and epithelial tissues (Amaroli et al., 2018).

The literature has clearly demonstrated that PBM has no deleterious effects on MSC (Kushibiki et al., 2015; Marques et al., 2016). However, the current research in literature has not provided a conclusive outcome (Marques et al., 2016) but it has only pointed out the effectiveness of the red wavelengths (Kushibiki et al., 2015; Marques et al., 2016). This evidence stresses the value of the results of our current study and the possible applications of our *in vivo* phototherapy investigations. In fact, the results of the most *in vitro* studies on PBM were obtained using wavelengths in the range 600–700 nm. However, through *in vivo* studies, the light-energy in this range of wavelengths can quickly disperse and does not penetrate to deeper target tissue layers in order to provide therapeutic effects. On the other hand, the wavelengths ranging from 800 up to 1100 nm which have a longer optical penetration depth can target deeper tissues (Avci et al., 2013; Pandeshwar et al., 2016). Therefore, the wavelengths in the range of 390–600 nm are used to treat *in vitro* culture or superficial tissues while the longer wavelengths in the range of 600–1100 nm are used to treat deeper-seated tissues. Moreover, the wavelengths in the range of 700–750 nm have been found to have a limited biochemical activity and therefore, they are not often used (Avci et al., 2013; Pandeshwar et al., 2016).

## Conclusion

In conclusion, our data prove for the first time that 808 nm diode laser irradiation, delivered by the flat-top hand-piece at the higher-fluence and -power of 64 J/cm2 and 1 W (CW) respectively, promotes BMSCs differentiation toward osteogenesis. Within the limits of our evaluation, our results suggest an additional possible laser effect based on its ability to increase TGFβ synthesis and to facilitate osteoblast differentiation by creating an anti-inflammatory effect on bone marrow stroma cells.

## Author contributions

AA, DA, VC, MS, FL, and SB: Conceived and designed the experiments; AA, DA, VC, MS, and FL: Performed the experiments, collected data and performed the data analyses; AA, DA, VC, MS, FL, and SB: Provided analysis tools; AA, DA, VC, MS, FL, RH, and SB: Wrote the manuscript.

### Conflict of interest statement

The authors declare that the research was conducted in the absence of any commercial or financial relationships that could be construed as a potential conflict of interest.
